# Supplementary material for: A Systematic Review of Pneumonitis Following Treatment with Immune Checkpoint Inhibitors and Radiotherapy
Source: Biomedicines. 2025 Apr 12;13(4):946. doi: 10.3390/biomedicines13040946 (PMC12025308; doi:10.3390/biomedicines13040946)
Supplement: Supplementary file 1 [file biomedicines-13-00946-s001.zip › biomedicines-3531029-supplementary.pdf]

Supplementary Figures of ‘Examining the Incidence and Characteristics of Pneumonitis Following Treatment with Immune Checkpoint Inhibitors and Radiotherapy’

No	Author, year	Design (prospective,retrospective, mixed)	Inclusion/Exclusion criteria clearly stated	Inclusion/Exclusion criteria reliably measured	Inclusion/Exclusion criteria uniformly applied	Same recruitment method across groups	Sample size sufficiently large (statistical power)	Specification of intervention exposure	Presesified outcomes	Appropriate selection of comparison group	Attempt to balance allocation	Adjustment for unintended exposure	Variation in execution of protocol	Outcome assessors blinding	Valid and reliable measurement of intervention/exposure	Valid and reliable measurement of outcome	Same length of follow-up	Sufficient follow-up	High attrition	Difference between groups in attrition	Baseline difference between groups controlled	Measurement of confounding variables reliable	Confounding variables in design/analysis	Sensitivity analysis for loss to follow-up	Missing data for primary outcomes	Appropriate statistical methods	Appropriate interpretation	Funding
1	Shaverdian N (2020) [11]	Retro	Partially	Yes	CD	CD	No	Medium	Partially	Yes	No	No	CD	No	Yes	CD	CD	Yes	CD	CD	No	CD	No	No	CD	Partially	Partially	Yes
2	Saito S (2021) [49]	Retro	Partially	Yes	Yes	CD	No	High	Yes	CD	No	Partially	CD	No	Yes	Yes	Yes	Yes	CD	CD	No	Yes	Yes	No	CD	Yes	Partially	No
3	Landman Y (2012) [19]	Retro	Yes	Partially	Yes	Yes	Partially	High	Yes	Partially	No	CD	Partially	No	Yes	Yes	Yes	Yes	CD	CD	Partially	Partially	Yes	No	CD	Yes	Yes	No
4	Jang JY (2021) [20]	Retro	Yes	Partially	Yes	Yes	Partially	Yes	Yes	Partially	No	CD	CD	No	Yes	Yes	Yes	Yes	CD	CD	Partially	Partially	Yes	No	CD	Yes	Yes	Yes
5	Barron F (2020) [21]	Retro	Yes	Yes	Yes	Yes	Partially	Yes	Yes	Yes	No	CD	CD	No	Yes	Yes	Yes	Yes	CD	CD	Partially	Partially	Yes	No	CD	Yes	Yes	Yes
6	Chen Y (2021) [40]	Mixed	Yes	Yes	Yes	Yes	Partially	Yes	Yes	Yes	No	CD	Partially	No	Yes	Yes	Yes	Yes	CD	CD	Partially	Partially	Yes	No	CD	Yes	Yes	Yes
7	Bi J (2022) [54]	Retro	Yes	Partially	Yes	Yes	Partially	Yes	Yes	Partially	No	No	Yes	No	Yes	Yes	Yes	Yes	CD	CD	Partially	Partially	Partially	No	CD	Yes	Yes	Yes
8	Sugimoto T (2021) [22]	Mixed	Yes	Yes	Yes	Yes	Partially	Yes	Yes	Yes	CD	No	Partially	No	Yes	Yes	Yes	Yes	No	No	Partially	Partially	Yes	CD	CD	Yes	Yes	Yes
9	Cousin F (2021) [23]	Retro	Yes	Yes	Yes	Yes	Partially	Yes	Yes	Partially	No	No	Partially	No	Yes	Yes	Partially	Partially	CD	CD	Partially	Partially	Yes	No	CD	Yes	Yes	No
10	Lu X (2022) [50]	Retro	Yes	Yes	Yes	Yes	Yes	Yes	No	Yes	No	Partially	Yes	No	Yes	Yes	Yes	Yes	CD	CD	Partially	Yes	Yes	No	CD	Yes	Yes	Yes
11	Thomas HM (2022) [18]	Retro	Yes	Yes	Yes	Yes	Yes	Yes	Yes	Yes	No	Partially	Yes	No	Yes	Yes	Yes	Yes	CD	CD	Partially	Yes	Yes	No	CD	Yes	Yes	Yes

Supplementary Figures of ‘Examining the Incidence and Characteristics of Pneumonitis Following Treatment with Immune Checkpoint Inhibitors and Radiotherapy’

**12**	Noda-Narita S (2022) [25]	Retro	Yes	Yes	Yes	Yes	Partially	Yes	Yes	Yes	No	Partially	Yes	No	Yes	Yes	Yes	Yes	CD	CD	Partially	Yes	Yes	No	CD	Yes	Yes	Yes
**13**	Saade LJ (2023) [26]	Retro	Yes	Yes	Yes	Yes	Partially	Yes	Yes	Yes	No	Partially	Yes	No	Yes	Yes	Yes	Yes	CD	CD	Partially	Yes	Yes	No	CD	Yes	Yes	CD
**14**	Neibart SS (2023) [27]	Retro	Yes	Yes	Yes	Yes	Yes	Yes	Yes	Yes	No	Partially	Yes	No	Yes	Yes	Yes	Yes	CD	CD	Partially	Yes	Yes	No	CD	Yes	Yes	Yes
**15**	Gao Y (2023) [51]	Retro	Yes	Yes	Yes	Yes	Partially	Yes	Yes	Yes	No	No	Yes	No	Yes	Yes	No	Partially	CD	CD	Partially	Yes	Partially	No	CD	Yes	Yes	Yes
**16**	Altan M (2023) [28]	Retro	Yes	Yes	Yes	Yes	Partially	Yes	Yes	Yes	No	No	Yes	No	Yes	Yes	No	Partially	CD	CD	Partially	Yes	Partially	No	CD	Yes	Yes	Yes
**17**	Preti BTB (2023) [29]	Retro	Yes	Yes	Yes	Yes	Yes	Yes	Yes	CD	No	No	Yes	No	Yes	Yes	Yes	Partially	CD	CD	CD	Yes	Partially	No	CD	Yes	Yes	No
**18**	Cai Z (2023) [30]	Retro	Yes	Yes	Yes	Yes	Partially	Yes	Yes	Yes	No	No	Yes	No	Yes	Yes	No	Partially	CD	CD	Partially	Yes	Partially	No	CD	Yes	Yes	Yes
**19**	Bi J (2024) [47]	Retro	Yes	Yes	Yes	Yes	Partially	Yes	Yes	CD	No	No	Yes	No	Yes	Yes	No	Partially	CD	CD	CD	Yes	Partially	No	CD	Yes	Yes	Yes
**20**	Yang Y (2023) [52]	Retro	Yes	Yes	Yes	Yes	Partially	Yes	Yes	Yes	Yes	No	Yes	No	Yes	Yes	No	Partially	CD	CD	Partially	Yes	Partially	No	CD	Yes	Yes	Yes
**21**	Lv X (2024) [31]	Retro	Yes	Yes	Yes	Yes	Partially	Yes	Yes	Yes	No	Partially	Yes	No	Yes	Yes	Yes	Yes	CD	CD	Partially	Yes	Yes	No	CD	Yes	Yes	Yes
**22**	Smesseim I (2024) [32]	Retro	Yes	Yes	Yes	Yes	Partially	Yes	Yes	Yes	No	Partially	Yes	No	Yes	Yes	Yes	Yes	CD	CD	Partially	Yes	Yes	No	CD	Yes	Yes	No
**23**	Kraus KM (2024) [33]	Retro	Yes	Yes	Yes	Yes	Partially	Yes	Yes	Yes	No	Partially	Yes	Yes	Yes	Yes	Yes	Yes	CD	CD	Partially	Yes	Yes	No	CD	Yes	Yes	Yes
**24**	Yamanaka Y (2024) [34]	Mixed	Yes	Yes	Yes	Yes	Partially	Yes	Yes	Yes	No	Partially	Yes	No	Yes	Yes	Yes	Partially	Yes	Yes	Partially	Yes	Yes	No	CD	Yes	Yes	Yes
**25**	Wang K (2024) [48]	Retro	Yes	Yes	Yes	Yes	Yes	Yes	No	Yes	No	Partially	Yes	No	Yes	Yes	Yes	Yes	CD	CD	Partially	Yes	Yes	No	CD	Yes	Yes	Yes
**26**	Song Z (2025) [53]	Retro	Yes	Yes	Yes	Yes	Partially	Yes	Yes	Yes	No	Partially	Yes	No	Yes	Yes	Yes	Yes	CD	CD	Partially	Yes	Yes	No	CD	Yes	Yes	Yes
**27**	Tjong M.C. (2022) [24]	Retro	Yes	Yes	Yes	Yes	Partially	Yes	Yes	Yes	No	No	Yes	No	Yes	Yes	Partially	Yes	CD	CD	No	Partially	Partially	No	CD	Yes	Yes	No
**28**	Murata S (2024) [35]	Retro	Yes	Yes	Yes	Yes	Yes	Yes	Yes	Yes	No	No	Yes	No	Yes	Yes	Partially	Yes	CD	CD	No	Partially	Partially	No	CD	Yes	Yes	No

**29**	Jung HA (2020) [36]	Retro	Yes	Yes	Yes	Yes	Partially	Yes	Yes	Yes	No	No	Yes	No	Yes	Yes	Partially	Yes	CD	CD	No	Partially	Partially	No	CD	Yes	Yes	Yes
**30**	Chu CH (2020) [37]	Retro	Yes	Yes	Yes	Yes	Partially	Yes	Yes	Yes	Partially	Yes	Yes	No	Yes	Yes	Partially	Yes	CD	CD	Yes	Yes	Yes	No	CD	Yes	Yes	Yes
**31**	Miura Y (2020) [38]	Retro	Yes	Yes	Yes	Yes	Partially	Yes	Yes	Yes	Partially	Yes	Yes	No	Yes	Yes	Partially	Yes	CD	CD	Yes	Yes	Yes	No	CD	Yes	Yes	No
**32**	Amino Y (2020) [39]	Retro	Yes	Yes	Yes	Yes	Partially	Yes	Yes	Yes	Partially	Yes	Yes	No	Yes	Yes	Partially	Yes	CD	CD	Yes	Yes	Yes	Yes	CD	Yes	Yes	Yes
**33**	Chen D (2020) [40]	Retro	Yes	Yes	Yes	Yes	Partially	Yes	Yes	Yes	Partially	Yes	Yes	No	Yes	Yes	Partially	Yes	CD	CD	Yes	Yes	Yes	Yes	CD	Yes	Yes	Yes
**34**	Innue H (2020) [41]	Retro	Yes	Yes	Yes	Yes	Partially	Yes	Yes	Yes	Partially	Yes	Yes	No	Yes	Yes	Partially	Yes	CD	CD	Yes	Yes	Yes	Yes	CD	Yes	Yes	Yes
**35**	Saad A (2022) [42]	Retro	Yes	Yes	Yes	Yes	Partially	Yes	Yes	Yes	No	Yes	Yes	No	Yes	Yes	Partially	Yes	CD	CD	Yes	Yes	Yes	Yes	CD	Yes	Yes	Yes
**36**	Wang CC (2021) [43]	Retro	Yes	Yes	Yes	Yes	Partially	Yes	Yes	Yes	No	Yes	Yes	No	Yes	Yes	Partially	Yes	CD	CD	Yes	Yes	Yes	Yes	CD	Yes	Yes	Yes
**37**	Desilets A (2020) [44]	Retro	Yes	Yes	Yes	Yes	Yes	Yes	Yes	Yes	No	Yes	Yes	No	Yes	Yes	Partially	Yes	CD	CD	Yes	Yes	Yes	Yes	CD	Yes	Yes	Yes
**38**	Tsukita Y (2021) [45]	Retro	Yes	Yes	Yes	Yes	Yes	Yes	Yes	Yes	No	Yes	Yes	No	Yes	Yes	Partially	Yes	CD	CD	Yes	Yes	Yes	Yes	CD	Yes	Yes	No
**39**	Avrillon V (2022) [46]	Retro	Yes	Yes	Yes	Yes	Yes	Yes	Yes	Yes	No	Yes	Yes	No	Yes	Yes	Partially	Yes	CD	CD	Yes	Yes	Yes	Yes	CD	Yes	Yes	Yes
**40**	Durm GA (2020) [56]	Pros	Yes	Yes	Yes	Yes	Yes	Yes	Yes	No	Yes	Yes	No	No	Yes	Yes	Yes	Yes	CD	CD	Yes	Yes	Yes	Yes	CD	Yes	Yes	Yes
**41**	Perez AB (2020) [58]	Pros	Yes	Yes	Yes	Yes	Partially	Yes	Yes	No	CD	Yes	No	No	Yes	Yes	Yes	Yes	No	CD	Yes	Yes	Yes	Yes	No	Yes	Yes	Yes
**42**	Welsh JW (2020) [60]	Pros	Yes	Yes	Yes	CD	Partially	Yes	Yes	No	CD	Yes	No	No	Yes	Yes	Yes	Yes	No	CD	CD	Yes	Yes	Yes	No	Yes	Yes	Yes
**43**	Welsh JW (2020) [61]	Pros	Yes	Yes	Yes	CD	Partially	Yes	Yes	No	CD	Yes	No	No	Yes	Yes	Yes	Yes	No	CD	CD	Yes	Yes	Yes	No	Yes	Yes	Yes
**44**	Gerassimo M (2022) [62]	Pros	Yes	Yes	Yes	CD	Yes	Yes	Yes	No	CD	Yes	No	No	Yes	Yes	Yes	Yes	No	CD	CD	Yes	Yes	Yes	No	Yes	Yes	Yes
**45**	Peters S (2019) [57]	Pros	Yes	Yes	Yes	CD	Partially	Yes	Yes	No	CD	Yes	No	No	Yes	Yes	Yes	Yes	No	CD	CD	Yes	Yes	Yes	No	Yes	Yes	Yes

Supplementary Table S1: RTI Item Bank Assessment of Retrospective and Non-Randomized Prospective Trials (CD= Cannot determine)

Supplementary Figures of 'Examining the Incidence and Characteristics of Pneumonitis Following Treatment with Immune Checkpoint Inhibitors and Radiotherapy'

No	Author, year	Domain 1: Risk of bias arising from the randomization process	Domain 2: Risk of bias due to deviations from the intended interventions (effect of assignment to intervention)	Domain 3: Risk of bias due to missing outcome data	Domain 4: Risk of bias in measurement of the outcome	Domain 5: Risk of bias in selection of the reported result	Notes
1	**Vansteenkiste JF (2024) [55]**	Some concerns	Some concerns	High risk	High risk	Some concerns	
2	**Peter S (2022) [59]**	Some concerns	Some concerns	High risk	Some concerns	Some concerns	
3	**Antonia (2017) [14]**	Some concerns	Some concerns	High risk	Low risk	Some concerns	PACIFIC trial-Longer follow-up in Spigel DR (2022) PMID:35108059
4	**Kelly RJ (2021) [9]**	Some concerns	Low risk	Some concerns	Low risk	Some concerns	CheckMate 577

Supplementary Table S2: **Cochrane** *Risk of Bias Version 2 Assessment of Randomized Clinical Trials.*
